# Neurodevelopmental outcomes in preterm or low birth weight infants with germinal matrix-intraventricular hemorrhage: a meta-analysis

**DOI:** 10.1038/s41390-023-02877-8

**Published:** 2023-11-07

**Authors:** Meicen Zhou, Shaopu Wang, Ting Zhang, Surong Duan, Hua Wang

**Affiliations:** 1 Department of Pediatrics, West China Second University Hospital, Sichuan University, Chengdu, 610041 Sichuan Province China; 2 Key Laboratory of Birth Defects and Related Diseases of Women and Children, Ministry of Education, Sichuan University, Chengdu, 610041 Sichuan Province China; 3 Bingzhou Medical University, Bingzhou, 264003 China

## Abstract

**Background:**

This meta-analysis aimed to identify the near- and long-term neurodevelopmental prognoses of preterm or low birth weight (LBW) infants with different severities of intraventricular hemorrhage (IVH).

**Methods:**

Four databases were searched for observational studies that were qualified using the Newcastle-Ottawa Scale.

**Results:**

37 studies involving 32,370 children were included. Compared to children without IVH, children with mild IVH had higher incidences of neurodevelopmental impairment (NDI), cerebral palsy (CP), motor/cognitive delay, hearing impairment and visual impairment, as well as lower scores of the mental development index (MDI) and psychomotor development (PDI). Moreover, compared to mild IVH, severe IVH increased susceptibilities of children to NDI, motor delay, CP, hearing impairment and visual impairment, with worse performances in MDI, PDI, motor score and IQ. Mild IVH was not associated with seizures or epilepsy.

**Conclusions:**

Adverse neurodevelopmental outcomes positively associated with the occurrence and severity of IVH in preterm or LBW infants, providing evidence for counseling and further decisions regarding early therapeutic interventions.

**Impact:**

- Adverse neurodevelopmental outcomes later in life were closely associated with the occurrence and severity of IVH in preterm or LBW infants.
- Our results highlight the importance to make prediction of the neurodevelopmental outcomes of children born preterm or LBW with a history of IVH, which will guide affected parents when their children need clinical interventions to reach the full potential.
- We emphasize the importance of identifying specific developmental delays that may exist in children with IVH, providing detailed information for the development of comprehensive intervention measures.

## Introduction

Intraventricular hemorrhage (IVH) is a prevalent complication that occurs in infants born prematurely, particularly those with a gestational age (GA) less than 32 weeks (i.e., very preterm and extremely preterm birth) or a low birth weight (LBW) less than 2500 g.^1^ Mounting evidence has indicated a 10–20% occurrence of IVH in preterm infants born before the 30th week of gestation^2^ and 20–25% among very low birth weight infants.^3^ When a newborn weighs below 750 g, the occurrence rate of severe IVH can reach up to 35–45%.^2^ The advancements in clinical management enable an increase in the survival rate of preterm infants; meanwhile, more survivors have a high risk to experience IVH that can result in brain damage and long-term neurological consequences. Notably, the risk of IVH in infants rises as GA or birth weight decreases, and is negatively associated with the level of infant maturity.^4,5^

In 1978, Papile et al.^6^ proposed a grading system for germinal matrix hemorrhage and intraventricular hemorrhage (GMH-IVH) based on the severity assessed by the computed tomography, which has been widely used for decades by clinicians and researchers.^7^ According to this standard, mild IVH is defined as grade I or II IVH, while severe IVH is defined as grade III or IV IVH. All grades of IVH are related to negative outcomes, such as moderate-to-severe neurological developmental impairment,^8^ increased risk of cerebral palsy (CP),^9^ and epilepsy.^10^ A higher level of IVH severity correlates with a higher risk of adverse outcomes in children.^4^ In clinical practices, approximately 25–50% of preterm infants with mild hemorrhage, e.g., GMH-IVH, is asymptomatic but can be diagnosed during routine screening. Predicting the neurodevelopmental outcome of children who have experienced a history of IVH (particularly mild IVH) early in life has been a concern and is a research topic, which can facilitate the development of prevention or treatment strategies in early or later life to improve their health and quality of life in childhood, school-age, and adolescent/adult.^11^ Although the relationship between severe IVH and negative neurodevelopmental outcomes has been discovered, whether and how mild IVH is associated with neurodevelopment later in life remains unclear. Numerous studies have attempted to address this issue by reporting prognostic outcomes from comparisons between children with mild IVH and those without IVH. However, conflicting findings have been observed regarding the neurological prognosis of children with mild IVH. Payne et al.^12^ reported no evidence relating mild-grade IVH to poorer neurodevelopmental outcomes at 18–24 months of age. Contrastingly, Klebermass-Schrehof et al.^13^ found a significantly higher rate of impairments, including CP and visual impairment, in children with mild IVH than in children without IVH. Bolisetty et al.^14^ observed that even children with mild IVH exhibited an increased incidence of neurosensory impairment, developmental delay, CP, and deafness at 2–3 years of corrected age. Vohr et al.^11^ showed that discrepancies among these results could be attributed to variations in the study design, population race, ethnicity, age, and birth weight. Moreover, the impacts of IVH transformed from mild to severe remains unclear. For infants who have already experienced premature birth and LBW, many are accompanied by the occurrence of IVH. It has been increasingly recognized that our knowledge of the differences in outcomes between different severities of IVH is vital for prognostic counseling and further decisions on early therapeutic interventions, in particular for children at the stage of neural plasticity in the developing brain.^15^

Systematic evaluations and meta-analyses of the neurological prognosis of IVH in children were conducted in 2015,^16^ 2022^17^ and 2023,^18^ which had shown evidences for neurodevelopmental impairment after mild and severe IVH. People usually use MDI and PDI to evaluate early cognitive and motor outcomes, because Bayley Scales of Infant and Toddler Development (BSID) is the gold standard for diagnosing early neurodevelopment. However, as a composite indicator, lower MDI may be associated with language or cognitive development. MDI and PDI lack the ability to explore delays in specific domains (motor/language/cognition), which is crucial for determining appropriate early interventions. Bayley Scales of Infant and Toddler Development III (Bayley III) avoided mutual evaluation between different domains to be more targeted. Moreover, there’s no effective meta-analysis for the comparison between mild and severe IVH. Consequently, this study aimed to summarize all available evidence across multiple databases on the neurodevelopmental outcomes of survivors born preterm or with LBW after experiencing IVH early in life, and categorized IVH into different degrees of severity where possible for detailed comparisons.

## Methods

The study was conducted according to the Preferred Reporting Items for Systematic Reviews and Meta-Analysis (PRISMA) guideline 2020, and is reported according to the PRISMA checklist (Table S1. PRISMA checklist). The meta-analysis has been registered on the online database PROSPERO (International Prospective Register of Systematic Reviews) with registration number CRD42023428828.

### Search strategy

We searched the following electronic databases: PubMed, Embase, Cochrane Library, and Web of Science, without language and data restrictions. We searched the databases from their inception until May 25, 2023. We utilized specific keywords, such as “Infant, Premature”, “Infant, Low Birth Weight”, and “Cerebral Intraventricular Hemorrhage”, and additional details regarding our search strategies are presented in Supplemental Material 1.

### Inclusion and exclusion criteria

#### Inclusion criteria

1. Patients: The study only considered patients who had been diagnosed with IVH through imaging methods such as transcranial ultrasound or magnetic resonance imaging. Studies that classified IVH based on the modified Papile criteria were included, and the details were as follows: grade I, subependymal bleeding limited to the germinal matrix; grade II, IVH extending into normal-sized ventricles and typically filling < 50% of the ventricular lumen; grade III, IVH extending into dilated ventricles; and grade IV, IVH with parenchymal extension.^6^ Other criteria that provided adequate information to classify IVH grade were also included. GA and birth weight: Only$<$37 weeks’ completed GA or birth weight $<$2500 g were included.
2. Type of studies: Observation studies, including cohort and case-control studies were included.
3. Outcomes: Studies that reported neurodevelopmental outcomes were included. Outcomes that satisfied at least one of the following criteria were included:

#### Primary outcomes

- Neurodevelopmental impairment (NDI): defined as the presence of any of the following: cognitive delay (scores on standardized cognitive tests that were one standard deviation (SD) below the mean or corresponded to scores ≤ 85 on the cognitive scale of the (BSID), moderate to severe CP (defined as a score of ≥ 2 on the Gross Motor Functional Classification System), severe hearing impairment (defined as a requirement of unilateral/bilateral hearing aids or cochlear implants; or severe visual impairment, defined as visual acuity ≤ 20/200 (metric scale) in the better eye with best conventional correction.

#### Secondary outcomes

- Mental Developmental Index (MDI): assessed by the BSID, the first and second editions were eligible.
- Psychomotor Developmental Index (PDI): assessed by the BSID, the first and second editions were eligible.
- Motor/cognitive score: assessed by the Bayley III or other scales that provided adequate information.
- IQ: assessed by the Stanford-Binet Intelligence Scale or other scales that provided adequate information.
- CP: as defined above.
- Seizures: clear records of seizures beyond the neonatal period and the antiepileptic medications used for seizure control were included. Related seizure types included single episodes, febrile seizures, and multiple seizure events.
- Epilepsy: defined as at least two unprovoked seizures occurring > 24 h apart or one seizure with a relevant abnormal electroencephalographic pattern or brain scan, suggesting a high probability of a second seizure.

#### Exclusion criteria

1. Studies that were duplicates or inaccessible for full-text review were excluded.
2. Non-human studies, letters/editorials, case reports, case series, conference abstracts, and meta-analyses were excluded.
3. Patients without a clear distinction of ages and birth weight were excluded.
4. Comparison: studies reported outcomes that compared whether IVH occurs or not and compared severe IVH and without IVH were excluded.

### Data extraction

The results of the four databases were imported into EndnoteX9 and initially integrated. Two authors (M.Z. and T.Z.) independently screened the studies for eligibility based on the inclusion criteria using titles and abstracts, with any disagreements resolved by a third party (S.D.). The second round of inclusion was based on a full-text screening. Additionally, the studies identified from the references were screened for eligibility.

We extracted the following data from each study: citation information, study type, country, the number of patients recruited and center conducted, maximum GA and maximum birth weight included in the study subjects, diagnosis used for IVH, comparison, and specific outcomes. S.D. checked the extracted data for accuracy and completeness. We resolved discrepancies through discussion and consulted the primary studies. If the complete data was not available from the study, we attempted to reach out to the corresponding author to obtain the missing information.

### Quality assessment

Methodological quality was assessed using the Newcastle-Ottawa Scale (NOS) for cohort or case-control studies, with the following domains evaluated: selection, comparability, and outcome (cohort studies) or exposure (case-control studies). This scale uses a rating system ranging from 0–9 points, with quality assessed as follows: low quality = 0–3; moderate quality = 4–6; and high quality = 7–9. Two authors (M.Z. and T.Z.) evaluated the assessment independently, and any disagreements were resolved by a third author (S.D.).

### Statistical analysis

We combined and analyzed the studies using STATA 15.0. Effect sizes were reported as odds ratio (OR) and 95% confidence intervals (CI) for dichotomous data and as standardized mean difference (SMD)/weighted mean difference (WMD) and 95% CI for continuous outcomes. For continuous outcomes, the study was included only when raw data were provided as mean and standardized difference (SD). I^2^ and Q tests were used to test for heterogeneity, with a significance level of *P* < 0.1 and I^2^ greater than 50%, indicating significant heterogeneity. When significant heterogeneity was observed, a random-effects model was used. Otherwise, a fixed-effects model was used. We conducted sensitivity analyses for each outcome by using a leave-one-study-out analysis(LOSO) whenever possible. Egger’s test was utilized to analyze publication bias for outcomes reported in at least 10 studies. When there was publication bias, the trim method was used to evaluate the impact of publication bias on the results. Statistical significance was defined as a two-tailed *P* < 0.05.

## Results

### Description of studies

**Fig. 1:**
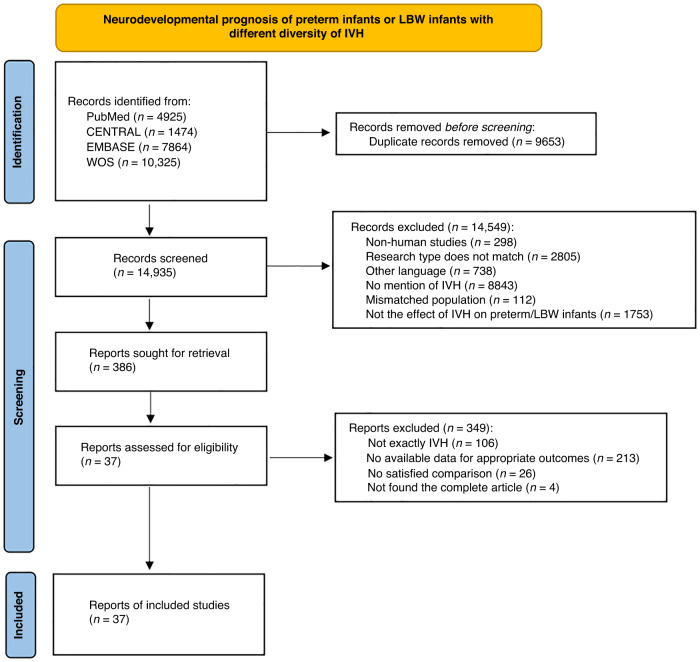
PRISMA flow diagram describing study selection for this meta-analysis. Each rectangle represents a step in the literature screening process. The left column represents the number of papers screened at each step, and the right side states the number of documents excluded and the reasons for exclusion.

Out of the total of 24,588 citations that were found through systematic research, 386 were considered suitable for a complete review. After applying our inclusion criteria, only 37^8–10,12–14,19–49^ studies were selected for this meta-analysis. These 37 studies comprised 34 cohort and three case-control studies. Figure 1 shows a PRISMA flow diagram of the search. Supplementary Table S2 presents the baseline characteristics of the 37 studies, including 32,370 children whose neurodevelopmental outcomes were evaluated from 6 months to 18 years of corrected age.

### Quality assessment

The risk of bias was assessed at the study level using an adapted version of the NOS, and the results are listed in Supplementary Table (Table S3: cohort study, Table S4: case-control study). All studies were rated above six points, which were classified as high quality, and could be deemed eligible for meta-analysis.

### Neurodevelopmental outcomes

#### Primary outcomes

##### NDI

**Fig. 2:**
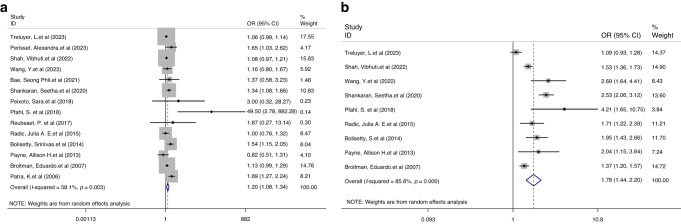
Forest plot showing the outcomes of NDI. **a** Comparison results between children with mild IVH vs. without IVH. **b** Comparison results between children with severe IVH vs. mild IVH.

14 studies presented the data on the outcome of NDI in the context of comparing the children with mild IVH to those without IVH. A random-effects model was applied as the heterogeneity of NDI between studies was significant (I^2^ = 58.1%, *P* = 0.003). Compared to the children without IVH, children born preterm or LBW with mild IVH had a significantly higher risk of NDI (OR 1.20, 95% CI 1.08, 1.34, *P* = 0.001, Fig. 2a). Furthermore, preterm or LBW children with severe IVH experienced a significantly higher rate of NDI than children with mild IVH (OR 1.78, 95% CI 1.44, 2.20, *P* < 0.001, Fig. 2b). The random-effects model was used due to the significant (I^2^ = 85.6%, *P* < 0.001) heterogeneity between studies.

### Secondary outcomes

#### Neuropsychomotor development

**Fig. 3:**
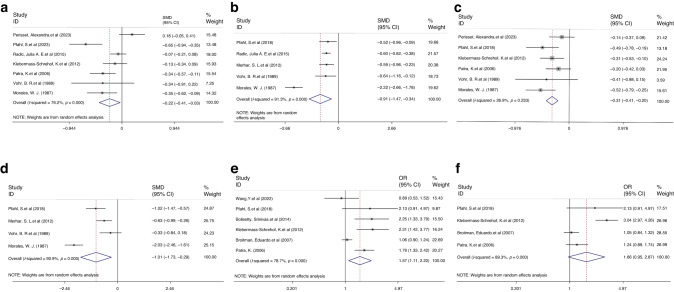
Forest plot showing the outcomes of MDI and PDI. **a** Mean difference of MDI for children with mild IVH vs. children without IVH. **b** Mean difference of MDI for children with severe IVH vs. children with mild IVH. **c** Mean difference of PDI for children with mild IVH vs. children without IVH. **d** Mean difference of PDI for children with severe IVH vs. children with mild IVH. **e** OR for the outcome of MDI scored below 70 for mild IVH vs. without IVH. **f** OR for the outcome of PDI scored below 70 for mild IVH vs. without IVH.

The heterogeneity of the mean MDI scores was significant in both comparisons (mild vs. no IVH: I^2^ = 76.2%, *P* < 0.001; severe vs. mild IVH: I^2^ = 91.3%, *P* < 0.001), and the random-effects models were thus used for both. Children with mild or severe IVH had significantly lower MDI scores than those without (SMD = −0.22, 95% CI −0.41, −0.03, *P* = 0.023, Fig. 3a) or with mild (SMD = −0.91, 95% CI −1.47, −0.34, *P* = 0.002, Fig. 3b) IVH.

Regarding the psychomotor development of children born preterm or LBW reflected by the PDI, we observed that children with mild or severe IVH had significantly lower means PDI scores than those without (SMD = −0.31, 95% CI −0.41, −0.30, *P* < 0.001; fixed-effects model: I^2^ = 26.9%, *P* = 0.233) or with mild (SMD = −1.01, 95% CI −1.73, −0.29, *P* = 0.006; random-effects model: I^2^ = 90.9%, *P* < 0.001) IVH (Fig. 3c, d).

When further categorizing children born preterm or LBW into groups based on the MDI/PDI score (i.e., normal development: ≥ 85; mild impairment in development: 70–84; moderate to severe impairment in development: ≤ 70), children with mild IVH showed a higher risk of moderate to severe impairment in mental development (OR 1.57, 95% CI 1.11, 2.22, Fig. 3e), and a trend(*P* = 0.073) towards a higher risk of moderate to severe impairment in psychomotor development than children without IVH(OR 1.66, 95% CI 0.95, 2.87, Fig. 3f).

#### Motor/cognitive score

**Fig. 4:**
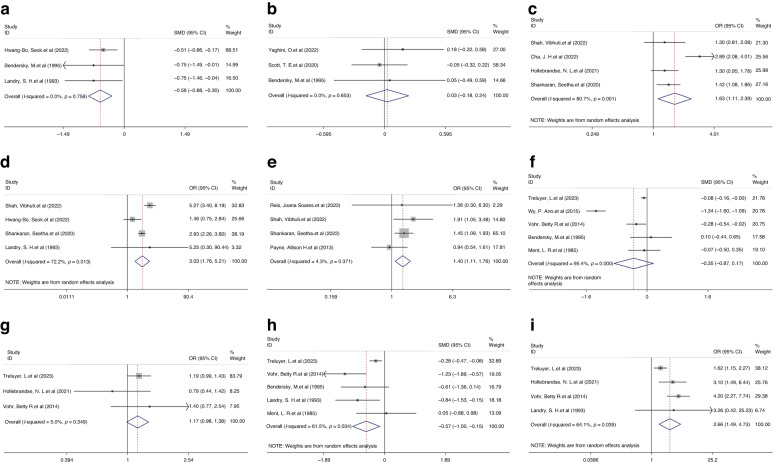
Forest plot showing the outcomes of motor scores, cognitive scores and IQ. **a** Mean difference of motor scores for children with severe IVH vs. children with mild IVH. **b** Mean difference of motor scores for children with mild IVH vs. children without IVH. **c** OR for the outcome of motor delay comparison between mild IVH vs. without IVH. **d** OR for the outcome of motor delay comparison between severe IVH vs. mild IVH. **e** OR for the outcome of cognitive delay comparison between mild IVH vs. without IVH. **f** Mean difference of IQ for children with mild IVH vs. children without IVH. **g** OR for the outcome of IQ scored below 70 or ranked under -2SD for mild vs. without IVH. **h** Mean difference of IQ for children with severe IVH vs. children with mild IVH. **i** OR for the outcome of IQ scored below 70 or ranked under −2SD for severe vs. mild IVH.

The heterogeneity of motor score was non-significant in both comparisons (mild vs. no IVH: I^2^ = 0.0%, *P* = 0.653; severe vs. mild IVH: I^2^ = 0.0%, *P* = 0.758), hence, we used the fixed-effects model. Children with a history of severe IVH had significantly lower motor scores than those with mild IVH (SMD = −0.59, 95% CI −0.88, −0.30, *P* < 0.001; Fig. 4a), but not for the comparison between children with a history of mild IVH and those without (SMD = 0.03, 95% CI −0.18, 0.24, *P* = 0.800; Fig. 4b).

The Bayley III motor/cognitive/language composite scores were deemed normalized to a mean 100, SD 15, and the presence of developmental delay was categorized as any delay above 2 SD. The subscale can be applied separately to identify a specific problem in children’s development. For motor score (mild vs. no IVH: four studies, I^2^ = 80.7%, *P* = 0.001; severe vs. mild IVH: four studies, I^2^ = 72.2%, *P* = 0.013), fixed-effects models were used. We observed an increased risk of motor delay when comparing children with mild IVH to those without IVH (OR 1.63, 95% CI 1.11, 2.39, *P* = 0.001; Fig. 4c), and a corresponding increase in risk of motor delay was also observed when comparing children with severe and mild IVH (OR 3.03, 95% CI 1.76, 5.21, *P* = 0.013; Fig. 4d).

Meanwhile, we observed sufficient studies of cognitive delay comparing children with mild or without IVH using Bayley III. Heterogeneity was not significant (four studies, I^2^ = 4.3%, *P* = 0.371), thus a fixed-effects models were used. We found an increased risk of cognitive delay for the comparison between the mild and without IVH groups (OR 1.40, 95% CI 1.11, 1.76, *P* = 0.005; Fig. 4e).

#### IQ

For the comparison between the mild and no IVH groups, we observed non-significant differences between children with a history of mild IVH and those without (SMD = −0.35, 95% CI −0.87, 0.17, *P* = 0.183; random-effects model: I^2^ = 95.4%, *P* < 0.001; Fig. 4f), while a trend was observed for IQ < −2SD (equivalent to IQ < 70, OR 1.17, 95% CI 0.98, 1.38, *P* = 0.349; fixed-effects model: I^2^ = 5.0%, *P* = 0.349; Fig. 4g).

For the comparison between the severe and mild IVH groups, significant heterogeneity in IQ scores (I^2^ = 61.5%, *P* = 0.034) or IQ < −2SD (I^2^ = 64.1%, *P* = 0.039) were not found, and the random-effects model was utilized. Severe IVH was associated with significantly lower IQ scores (SMD = −0.57, 95% CI −1.00, −0.15, *P* = 0.008; Fig. 4h) than mild IVH in preterm or LBW children. Additionally, patients with severe IVH had a significantly higher risk of having an IQ score below 70 or below -2SD than those with mild IVH (OR 2.66, 95% CI 1.49, 4.73, *P* = 0.001, Fig. 4i).

#### Hearing impairment and visual impairment

**Fig. 5:**
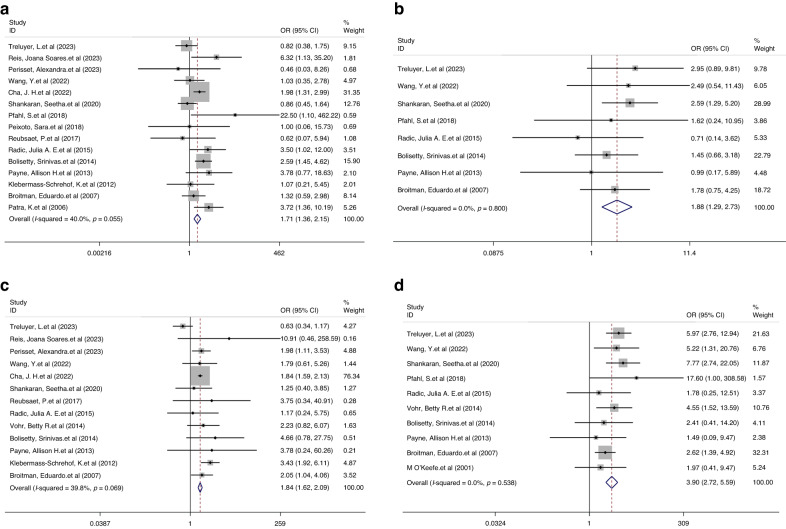
Forest plot showing the outcomes of hearing impairment and visual impairment. **a** OR for the outcome of hearing impairment comparison between mild IVH vs. without IVH. **b** OR for the outcome of hearing impairment comparison between severe IVH vs. mild IVH. **c** OR for the outcome of visual impairment comparison between mild IVH vs. without IVH. **d** OR for the outcome of visual impairment comparison between severe IVH vs. mild IVH.

For hearing impairment (mild vs. no IVH: I^2^ = 40.0%, *P* = 0.055; severe vs. mild IVH: I^2^ = 0.0%, *P* = 0.800) and visual impairment (mild vs. no IVH: I^2^ = 39.8%, *P* = 0.069; severe vs. mild IVH: I^2^ = 0.0%, *P* = 0.538), fixed-effects models were used because the heterogeneity was not significant in both comparisons. We observed a significantly higher risk of hearing impairment in children with a history of mild IVH than in those without IVH (OR 1.71, 95% CI 1.36, 2.15, *P* < 0.001, Fig. 5a), and a corresponding increased risk was also observed when comparing children with severe and mild IVH (OR 1.88, 95% CI 1.29, 2.73, *P* = 0.001; Fig. 5b).

Regarding visual impairment, we observed an increased risk when comparing children with mild IVH to those without IVH (OR 1.84, 95% CI 1.62, 2.09, *P* < 0.001; Fig. 5c), and a corresponding increase in risk was also observed when comparing children with severe and mild IVH (OR 3.90, 95% CI 2.72, 5.59, *P* < 0.001; Fig. 5d).

#### CP

**Fig. 6:**
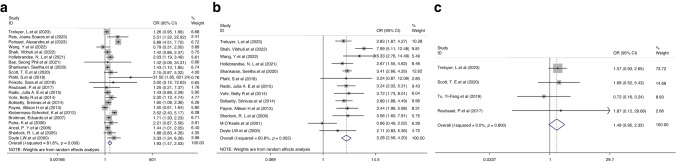
Forest plot showing the outcomes of CP and seizure events or epilepsy. **a** OR for the outcome of CP comparison between children with mild IVH vs. without IVH. **b** OR for the outcome of CP comparison between children with severe IVH vs. mild IVH. **c** OR for the outcome of seizure events or epilepsy for children with mild IVH vs. without IVH.

Significant heterogeneity was observed in the comparison between the mild and no IVH groups (I^2^ = 81.8%, *P* < 0.001) or between the severe and mild IVH groups (I^2^ = 60.8%, *P* = 0.002); therefore, random-effects models were utilized. Compared with those without IVH, children with a history of mild IVH had a significantly higher risk of CP (OR 1.93, 95% CI 1.47, 2.53; *P* < 0.001; Fig. 6a). Severe IVH was associated with a significantly higher risk of CP in children born preterm or LBW than in those with mild IVH (OR 3.28, 95% CI 2.56, 4.20, *P* < 0.001; Fig. 6b).

#### Seizure events or epilepsy

We found sufficient studies for the comparison between the mild and no IVH groups. No significant heterogeneity was observed in the comparison between the mild and no IVH groups across the studies (I^2^ = 0.0%, *P* = 0.800); thus, a fixed-effects model was applied. We did not find a trend in the increased risk of epilepsy when comparing children with mild IVH to those without IVH (OR 1.49, 95% CI 0.95, 2.33, *P* = 0.084, Fig. 6c).

### Publication bias and sensitivity analyses

We conducted sensitivity analyses for each correlation and comparison using a LOSO; however, no significant changes were observed in the relative risk for any comparison (Supplementary Fig. S1–6). We conducted an analysis to determine whether publication bias was present in the studies associated with CP (mild vs. no IVH: *P* = 0.934; severe vs. mild IVH: *P* = 0.645) and visual impairment (mild vs. no IVH: *P* = 0.658; severe vs. mild IVH: *P* = 0.928) in both comparisons, meanwhile, with NDI (*P* = 0.019) and hearing impairment (*P* = 0.825) for the comparison between the mild and no IVH groups. Egger’s test (Supplementary Fig. S7) revealed no evidence of potential publication bias among the trials included in the study, except for the outcome of NDI for the comparison between the mild and no IVH groups. Therefore, we used the trim method to evaluate the impact of publication bias on NDI, after incorporating data from 5 virtual studies, the meta-analysis was conducted again. We observed significant heterogeneity (*P* < 0.001), thus a random-effects model was used. The combined results were robust (OR 1.13, 95% CI 1.00, 1.27; *P* = 0.047; Supplementary Fig. S8), therefore, the existence of publication bias has no impact on our results.

## Discussion

In this study, we analyzed the impact of different severities of IVH in preterm or LBW infants on neural development during growth and revealed a positive correlation between different IVH severities and a series of adverse neurodevelopmental prognostic outcomes. We observed that children with mild IVH (compared to those without IVH) or severe IVH (compared to those with mild IVH) had an increased risk of NDI. Regarding secondary outcomes, mild IVH increased the risk of MDI, cognitive delay, PDI, motor delay, hearing and visual impairments, and CP; except for IQ, seizure events and epilepsy, compared with children without IVH. Severe IVH resulted in a worse prognosis than that of mild IVH in all secondary outcomes. Unfortunately, there were insufficient studies on seizure events or epilepsy analysis when comparing children with severe and mild IVH.

With more studies included in our analysis, our findings suggested that with an increase in the severity of IVH, the risk of NDI increased, leading to worse neurodevelopmental outcomes, which validates and expands the previous meta-analysis with higher confidence.^16,17^ Our results indeed provided the rationality that the variations in the occurrence of NDI might depend on the IVH severity since not all children with IVH will experience NDI. To further confirm the prognosis of neurodevelopmental outcomes, we explored the MDI and PDI scores of the children and found that children with mild or severe IVH in the two comparisons had significantly lower scores for both mental outcomes. To our knowledge, this is the first study to determine the differences in MDI and PDI scores in children with different severities of IVH. Based on the primary outcomes of NDI, MDI, and PDI, our study confirmed that mild IVH impacts the neurodevelopmental outcomes of preterm or LBW infants, which addresses the current contradictory findings to a certain degree through an unbiased systematic evaluation. These findings can help identify developmental delays of the brain or disabilities and encourage us to pay more attention to follow-ups for mild IVH.

To further explore the other critical impacts of different severities of IVH in preterm or LBW infants, we analyzed the neurodevelopmental outcomes from motor, cognitive, and sensory perspectives. Regarding motor scores, we found significantly lower scores in children with a severe IVH history than in those with a mild IVH history using different scales, while no association between mild IVH and motor scores was observed. Moreover, we explored the incidence of motor delay by using the Bayley III or clearly stated by the author, results showed that with an increased in the severity of IVH, children had a higher risk of motor delay. We also explored the fine and gross motor development by using the BSID-II for children with mild and no IVH, PDI < 70 was deemed moderate to severe motor impairment. The difference between the two groups were not significant.

From a cognitive perspective, we first observed that children with mild IVH had a higher risk of cognitive delay than children without IVH by using the Bayley III. Furthermore, we used IQ to revealed the long-term impact on cognitive domain varying severity IVH. We found a lower IQ in children with severe IVH than those with mild IVH, and children with severe IVH had a higher risk of scoring below 70 or ranking under −2SD in IQ. No association between mild IVH and IQ was observed neither specific scores nor ranking under −2SD, and we assumed that this was highly attributed to the fact that IQ is influenced by surrounding factors later in life as the child grows, such as the increasing influences of family, social, and environmental characteristics.^11^ These findings about motor and cognitive perspectives above are crucial because the related limitations are one of the main concerns of the parents when questioning clinicians about the child’s prognosis, and early intervention for preterm infants benefits both cognitive and motor functions during infancy and cognitive outcomes by preschool age.^50^ It is also vital to conduct long-term follow-ups with multiple intervening assessment points in the rehabilitation department to ensure a well-developed trajectory.

Regarding sensory impact, our study found an increased risk of hearing and visual impairment in children with mild IVH compared with those without IVH; meanwhile, an increased risks were also observed in children with mild or severe IVH. Visual impairment and hearing loss can negatively impact a child’s quality of life, physical well-being, and autonomy for the most basic activities, further increasing their dependence on a caregiver.^51^ These findings highlight the importance of arranging regular monitoring to assess the hearing and vision abilities of children born preterm or LBW with a history of IVH. This would help to identify and address impairments at an early stage. More importantly, our results indicate that for children with a history of IVH, follow-up should be conducted immediately after discharge and persist for at least 3 years to avoid inaccurate screening results and missing the optimal treatment time, which will significantly impact the quality of life of children.

Regarding sequelae, we focused on CP, seizure events or epilepsy. We found an association between increased severity of IVH and an increased risk of CP, which was reliable for both comparisons with low heterogeneity and without publication bias. Additionally, regarding seizure events or epilepsy, we observed no association between mild and no IVH groups. However, we could not conduct a quantitative analysis to determine whether there was a significant difference in outcomes between children with mild and severe IVH because of inadequate data. Although existing researches have shown that severe IVH is a risk factor for epilepsy.^52^ Tu et al.^10^ proposed that high-grade IVH may destroy the ventricular zone and predispose the immature brain to epileptogenic. Taking the previous findings and our results into account, we agree that severe IVH may increase the risk of seizure events or epilepsy in children born preterm or LBW. From this point of view, prevention and management should be individualized according to the severity of IVH, and specific attention should be paid to avoid the aggravation of IVH.

With the publication of Bayley III, people can better evaluate the specific developmental delays that may exist during the growth process of infants and young children. This ability is crucial for determining appropriate early intervention. To our knowledge, this is the first meta-analysis that confirmed IVH impacts on motor and cognitive domains mainly focused on bayley III. We provide a plethora of insights demonstrating the long-term impact of IVH on neurodevelopmental outcomes in children born preterm or LBW. These findings will be significant for healthcare providers and parents of affected children to provide knowledge about the areas where a child needs to be improved, and also serve as a reminder for medical professionals to identify and prevent the risk factors of IVH at an early stage. The most effective approach for controlling high-risk factors is to reduce the incidence of premature births or LBW. While sometimes premature birth or LBW cannot be avoided, the neonatal department must conduct early examinations to identify IVH. Moreover, an increase in the chance of the children who had IVH receiving early intervention services like rehabilitation exercises, even for children with mild IVH, will benefit them to reach their full potential. Study has shown evidences for neurodevelopmental impairment after severe IVH,^18^ and we confirmed the poor impacts of children with IVH transformed from mild to severe severity in all aspects, which were closely related to clinical practice, measures should be taken to prevent its occurrence or worsening severity.

This study has some limitations. Due to the limited number of included studies, the outcomes could not explain the source of heterogeneity across studies through traditional methods. Furthermore, qualified studies on different IVH severities remain limited, especially in seizure events or epilepsy, and we hope to conduct more research on this aspect to increase the evidence even more sufficiently.

## Conclusions

Overall, we reach a sound conclusion that different severities of IVH in infants are associated with multiple adverse neurodevelopmental outcomes later in life. Children with severe IVH had a higher risk of adverse neurodevelopmental outcomes among all the outcomes we assessed than children with mild IVH. Conversely, mild IVH was associated with an increased risk of NDI, MDI, PDI, motor/cognitive delay, hearing impairment, visual impairment, and CP compared with children without IVH. Notably, in high-risk populations, such as premature or LBW infants, attention should be paid to the occurrence of IVH as the early detection and treatment conducted promptly can avoid adverse outcomes later in life. Moreover, because mild IVH has been proven to be associated with poor neurological outcomes, healthcare professionals can counsel and educate family members about the potential risks and necessary precautions to be taken after discharge. Regarding the overall management of IVH, given the rapid development of the early nervous system in infants, tracking their neurodevelopmental outcomes is a continuous and long-term process. Multidisciplinary team collaboration should be adopted through healthcare efforts throughout the prenatal, neonatal, and childhood stages to allow children to reach their full potential in neurodevelopment, including cognition, motor, and sensation effects later in life.

### Supplementary information

Supplemental Material 1

## Data Availability

All data generated or analyzed during this study have been included in this published article (and its Supplementary Information files).
