# Supplementary material for: Neurodevelopmental outcomes in preterm or low birth weight infants with germinal matrix-intraventricular hemorrhage: a meta-analysis
Source: Pediatr Res. 2023 Nov 7;95(3):625–33. doi: 10.1038/s41390-023-02877-8 (PMC10899112; doi:10.1038/s41390-023-02877-8)
Supplement: Supplementary file 1 — Supplemental Material 1 [file 41390_2023_2877_MOESM1_ESM.pdf]

Supplemental Material 1 Search strategy

1. Search strategy of PubMed:

No.	Search items
#1	(((((((((((((Infant, Premature[MeSH Terms]) OR (Infant, Premature[Title/Abstract])) OR (Neonatal Prematurity[Title/Abstract])) OR (premature[Title/Abstract])) OR (premature baby[Title/Abstract])) OR (premature birth[Title/Abstract])) OR (premature infant[Title/Abstract])) OR (premature neonate[Title/Abstract])) OR (premature newborn[Title/Abstract])) OR (Premature Infant*[Title/Abstract])) OR (prematuritas[Title/Abstract])) OR (preterm baby[Title/Abstract])) OR (Preterm Infant*[Title/Abstract])) OR (preterm neonate[Title/Abstract])) OR (preterm newborn[Title/Abstract])
#2	(((((((((Infant, Low Birth Weight[MeSH Terms]) OR (Infant*, Low Birth Weight[Title/Abstract])) OR (LBW infant[Title/Abstract])) OR (LBW neonate[Title/Abstract])) OR (LBW newborn[Title/Abstract])) OR (low birth weight[Title/Abstract])) OR (Low Birth Weight Infant*[Title/Abstract])) OR (Low Birth Weights[Title/Abstract])) OR (low birthweight[Title/Abstract])) OR (neonatal underweight[Title/Abstract])
#3	(((((Intraventricular Hemorrhage[MeSH Terms]) OR (brain bleeding[Title/Abstract])) OR (brain haemorrhage*[Title/Abstract])) OR (brain hemorrhage*[Title/Abstract])) OR (Intraventricular Haemorrhage*[Title/Abstract])) OR (Intraventricular Hemorrhage*[Title/Abstract])
#4	(#1 OR #2) AND #3

2. Search strategy of Embase:

No.	Search items
#1	'infant, premature'/exp
#2	'infant, premature':ab,ti OR 'neonatal prematurity':ab,ti OR 'premature':ab,ti OR 'premature baby':ab,ti OR 'premature birth':ab,ti
#3	'premature infant':ab,ti OR 'premature neonate':ab,ti OR 'premature newborn':ab,ti OR 'premature infants':ab,ti OR 'prematuritas':ab,ti
#4	'preterm baby':ab,ti OR 'preterm infant':ab,ti OR 'preterm neonate':ab,ti OR 'preterm newborn':ab,ti
#5	#1 OR #2 OR #3 OR #4
#6	'infant, low birth weight'/exp
#7	'infant, low birth weight':ab,ti OR 'infants, low birth weight':ab,ti OR 'lbw infant':ab,ti OR 'lbw neonate':ab,ti OR 'lbw newborn':ab,ti
#8	'low birth weight':ab,ti OR 'low birth weight infant':ab,ti OR 'low birth weight infants':ab,ti OR 'low birth weights':ab,ti OR 'low birthweight':ab,ti
#9	'neonatal underweight':ab,ti
#10	#6 OR #7 OR #8 OR #9
#11	#5 OR #10
#12	'brain hemorrhage'/exp
#13	'brain hemorrhage':ab,ti OR 'brain hemorrhages':ab,ti OR 'brain bleeding':ab,ti OR 'brain haemorrhage':ab,ti OR 'brain haemorrhages':ab,ti

#14	'intraventricular haemorrhage':ab,ti OR 'intraventricular haemorrhages':ab,ti OR 'intraventricular hemorrhage':ab,ti OR 'intraventricular hemorrhages':ab,ti
#15	#12 OR #13 OR #14
#16	#11 AND #15

3. Search strategy of Cochrane Library

No.	Search items
#1	MeSH descriptor: [Infant, Premature] explode all trees
#2	("Infant, Premature" OR "Neonatal Prematurity" OR "premature" OR "premature baby" OR "premature birth"):ti,ab,kw (Word variations have been searched)
#3	("premature infant" OR "premature neonate" OR "premature newborn" OR "Premature Infants" OR "prematuritas"):ti,ab,kw (Word variations have been searched)
#4	("preterm baby" OR "Preterm Infant" OR "Preterm Infants" OR "preterm neonate" OR "preterm newborn"):ti,ab,kw (Word variations have been searched)
#5	#1 OR #2 OR #3 OR #4
#6	MeSH descriptor: [Infant, Low Birth Weight] explode all trees
#7	("Infant, Low Birth Weight" OR "Infants, Low Birth Weight" OR "LBW infant" OR "LBW neonate" OR "LBW newborn"):ti,ab,kw (Word variations have been searched)
#8	("low birth weight" OR "Low Birth Weight Infant" OR "Low Birth Weight Infants" OR "Low Birth Weights" OR "low birthweight"):ti,ab,kw (Word variations have been searched)
#9	(neonatal underweight):ti,ab,kw (Word variations have been searched)
#10	#6 OR #7 OR #8 OR #9
#11	#5 OR #10
#12	MeSH descriptor: [Cerebral Intraventricular Hemorrhage] explode all trees
#13	("brain bleeding" OR "brain haemorrhage" OR "brain haemorrhages" OR "brain hemorrhage" OR "brain hemorrhages"):ti,ab,kw (Word variations have been searched)
#14	("Intraventricular Haemorrhage" OR "Intraventricular Haemorrhages" OR "Intraventricular Hemorrhage" OR "Intraventricular Hemorrhages"):ti,ab,kw (Word variations have been searched)
#15	#12 OR #13 OR #14
#16	#11 AND #15

4. Search strategy of Web of Science

No.	Search items
#1	((((((((((((TS=(Infant, Premature)) OR TS=(Neonatal Prematurity)) OR TS=(premature)) OR TS=(premature baby)) OR TS=(premature birth)) OR TS=(premature infant)) OR TS=(premature neonate)) OR TS=(premature newborn)) OR TS=(Premature Infants)) OR TS=(prematuritas)) OR TS=(preterm baby)) OR TS=(Preterm Infant*)) OR TS=(preterm neonate)) OR TS=(preterm newborn)
#2	((((((((TS=(Infant*, Low Birth Weight)) OR TS=(LBW infant)) OR TS=(LBW neonate)) OR TS=(LBW newborn)) OR TS=(low birth weight)) OR TS=(Low Birth Weight Infant*)) OR TS=(Low Birth Weights)) OR TS=(low birthweight)) OR TS=(neonatal underweight)

#3	#1 OR #2
#4	((((TS=(brain bleeding)) OR TS=(brain haemorrhage*)) OR TS=(brain hemorrhage*)) OR TS=(Intraventricular Haemorrhage*)) OR TS=(Intraventricular Hemorrhage*)
#5	#3 AND #4

Supplementary Figures

Figure S1. Sensitivity analysis for the outcome of NDI. (a) Comparison results between children with mild IVH vs. without IVH. **(b)** Comparison results between children with severe IVH vs. mild IVH.

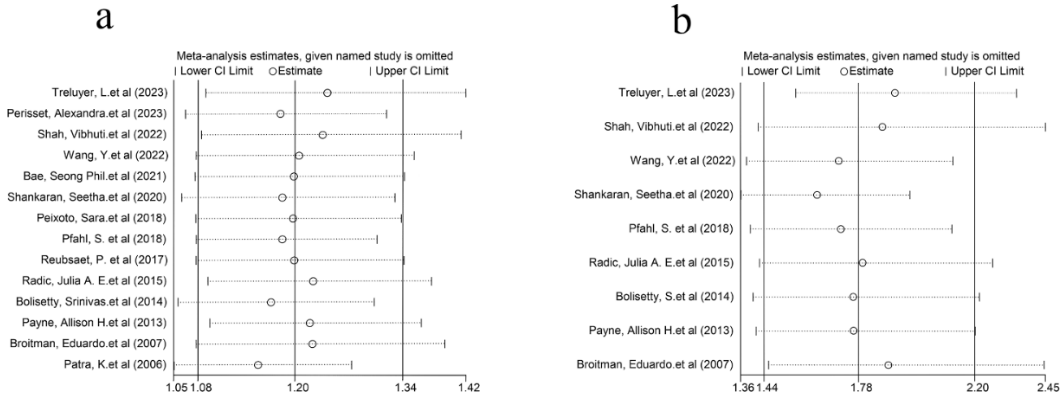

Figure S2. Sensitivity analysis for the outcome of MDI and PDI. (a) Mean difference of MDI for children with mild IVH vs. children without IVH. **(b)** Mean difference of MDI for children with severe IVH vs. children with mild IVH. **(c)** Mean difference of PDI for children with mild IVH vs. children without IVH. **(d)** Mean difference of PDI for children with severe IVH vs. children with mild IVH. **(e)** OR for the outcome of MDI scored below 70 for mild IVH vs. without IVH. **(f)** OR for the outcome of PDI scored below 70 for mild IVH vs. without IVH.

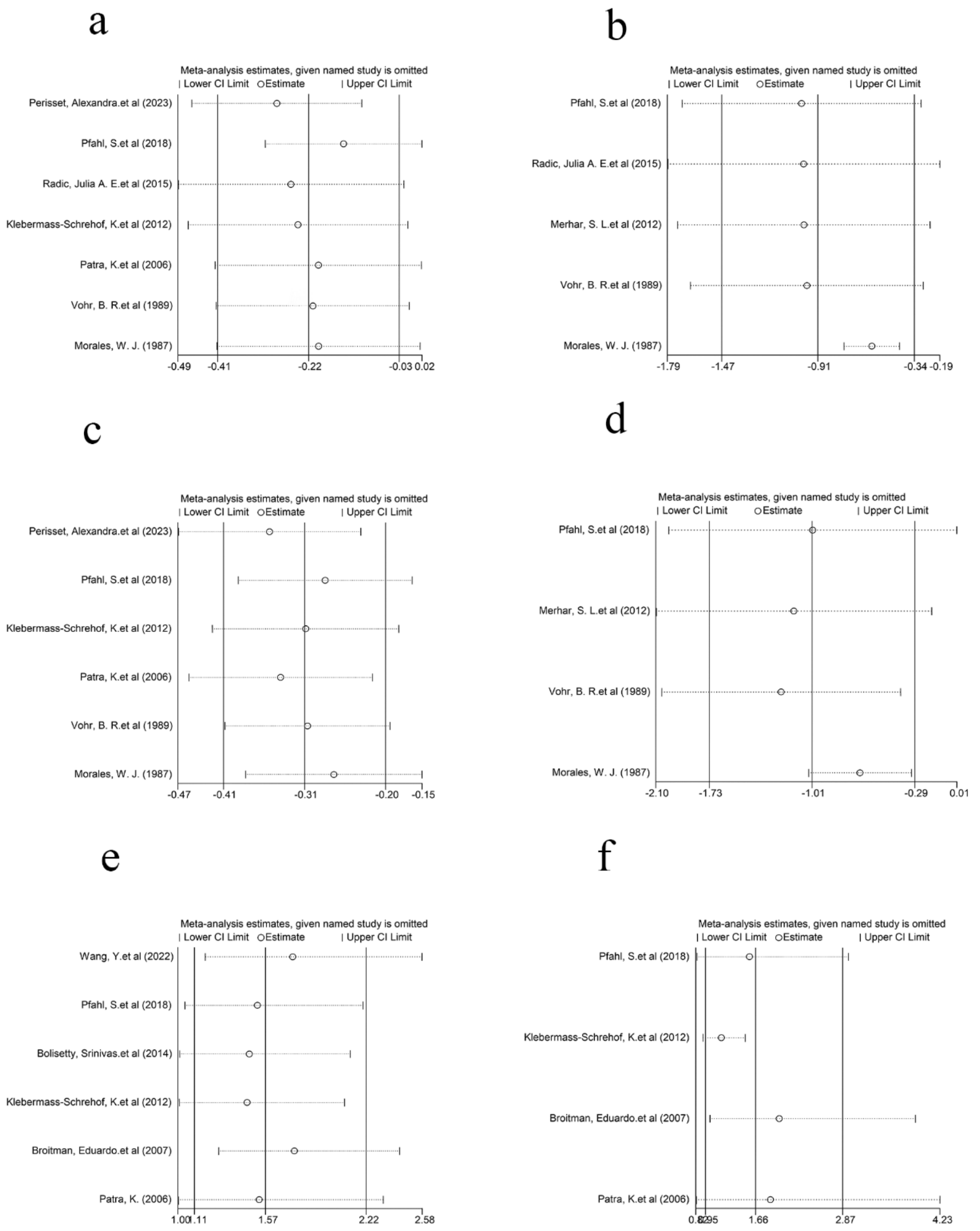

Figure S3. Sensitivity analysis for the outcome of motor scores. (**a**) Mean difference of motor scores for children with severe IVH vs. children with mild IVH. (**b**) Mean difference of motor scores for children with mild IVH vs. children without IVH. (**c**) OR for the outcome of motor delay comparison between mild IVH vs. without IVH. (**d**) OR for the outcome of motor delay comparison between severe IVH vs. mild IVH.

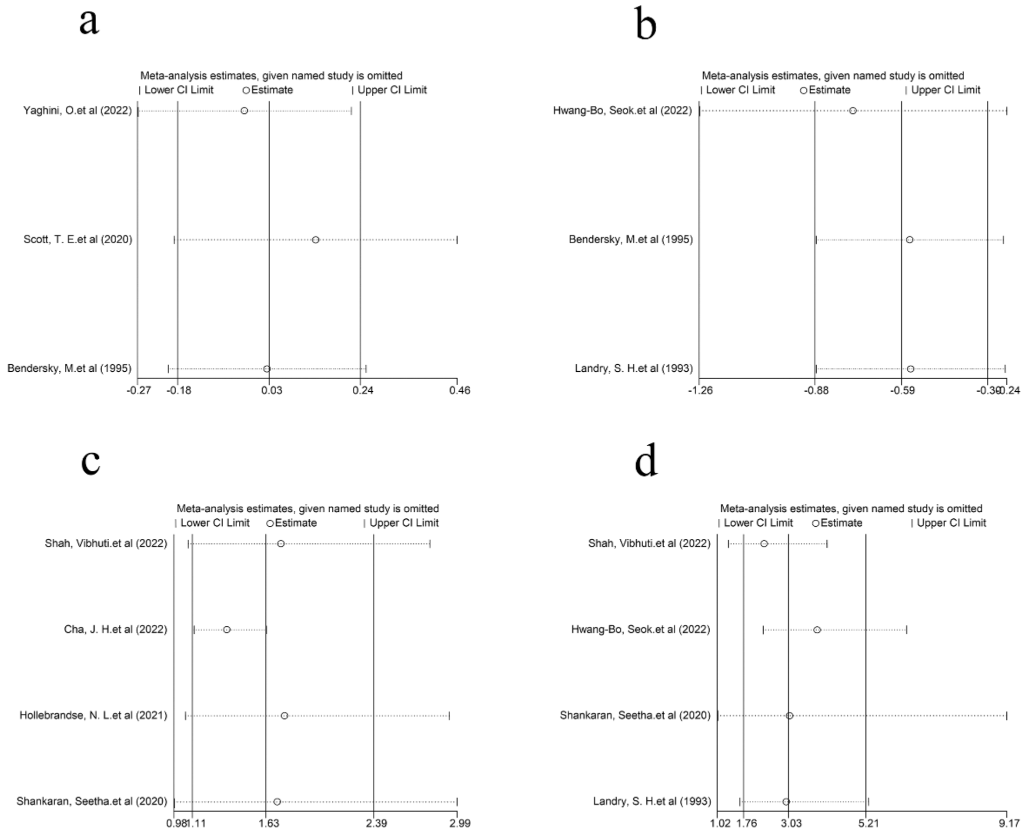

Figure S4. Sensitivity analysis for the outcome of cognitive score and IQ. (a) OR for the outcome of cognitive delay comparison between mild IVH vs. without IVH. **(b)** Mean difference of IQ for children with mild IVH vs. children without IVH. **(c)** OR for the outcome of IQ scored below 70 or ranked under -2SD for mild vs. without IVH. **(d)** Mean difference of IQ for children with severe IVH vs. children with mild IVH. **(e)** OR for the outcome of IQ scored below 70 or ranked under -2SD for severe vs. mild IVH.

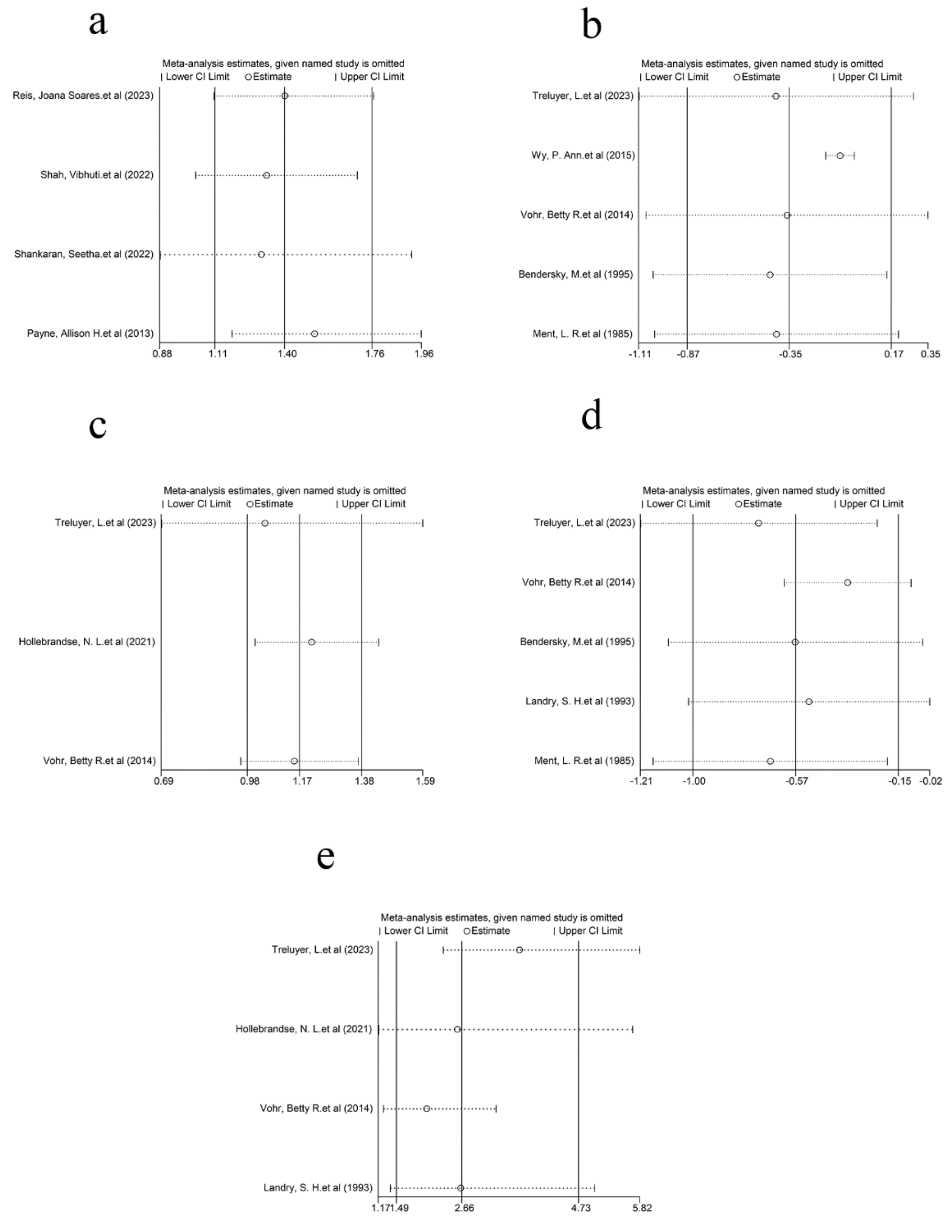

Figure S5. Sensitivity analysis for the outcome of hearing impairment and visual impairment. (a) OR for the outcome of hearing impairment comparison between mild IVH vs. without IVH. **(b)** OR for the outcome of hearing impairment comparison between severe IVH vs. mild IVH. **(c)** OR for the outcome of visual impairment comparison between mild IVH vs. without IVH. **(d)** OR for the outcome of visual impairment comparison between severe IVH vs. mild IVH.

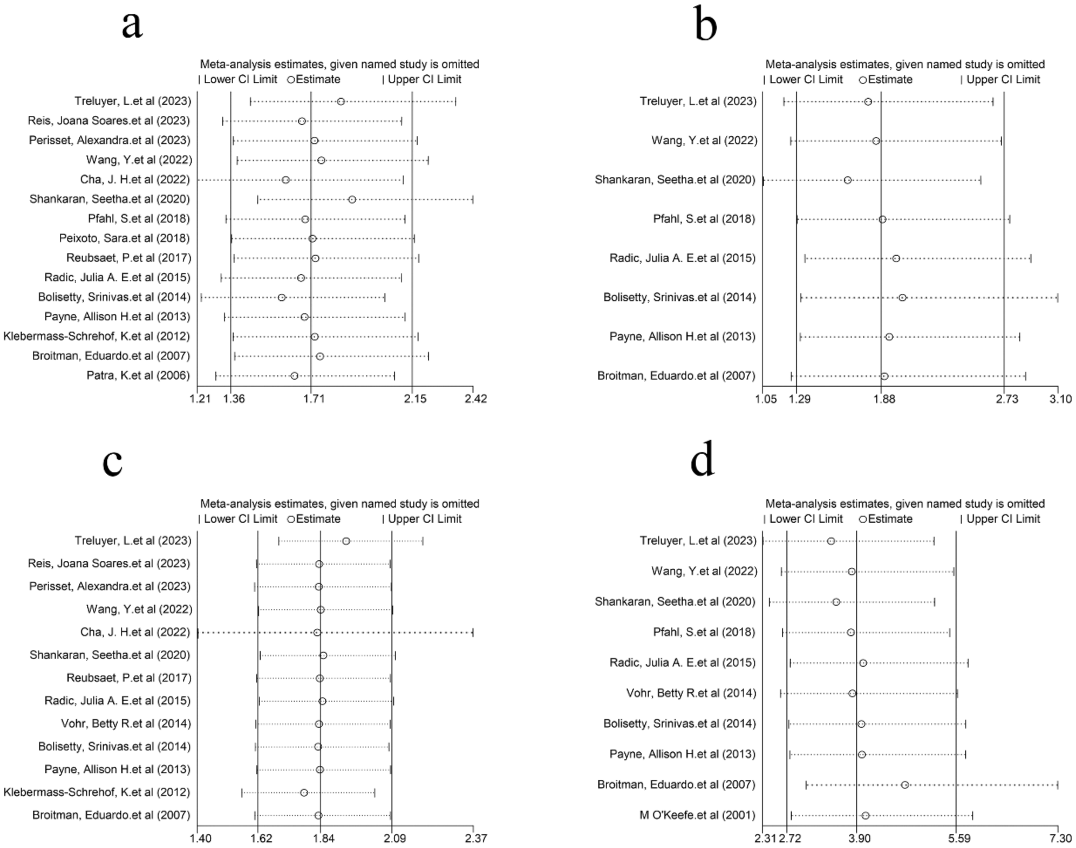

Figure S6. Sensitivity analysis for the outcome of CP and seizure events or epilepsy. (a) OR for the outcome of CP comparison between children with mild IVH vs. without IVH. **(b)** OR for the outcome of CP comparison between children with severe IVH vs. mild IVH. **(c)** OR for the outcome of seizure events or epilepsy for children with mild IVH vs. without IVH.

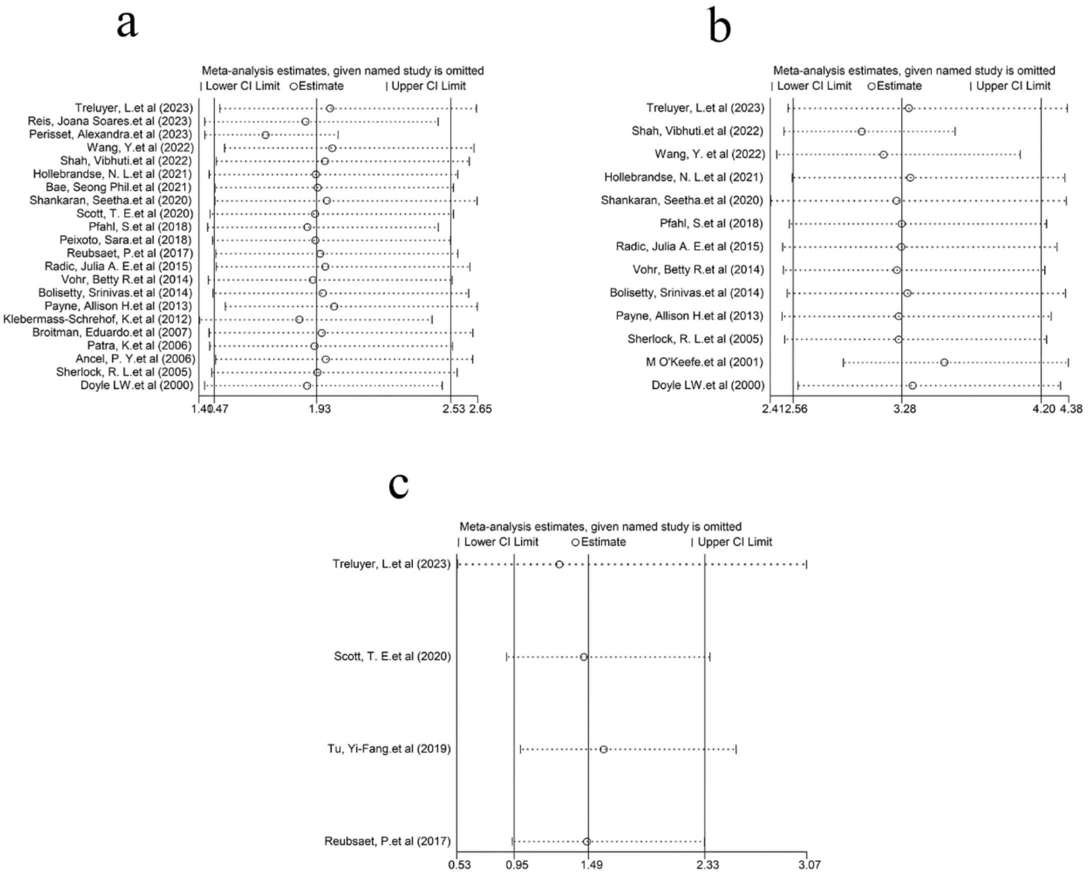

Figure S7. Publication bias plot of CP, visual impairment, NDI and hearing impairment. (a) CP for the comparison between children with mild IVH vs. without IVH. **(b)** CP for the comparison between children with severe IVH vs. mild IVH. **(c)** Visual impairment for the comparison between children with mild IVH vs. without IVH. **(d)** Visual impairment for the comparison between children with severe IVH vs. mild IVH. **(e)** NDI for the comparison between children with mild IVH vs. without IVH. **(f)** Hearing impairment for the comparison between children with mild IVH vs. without IVH.

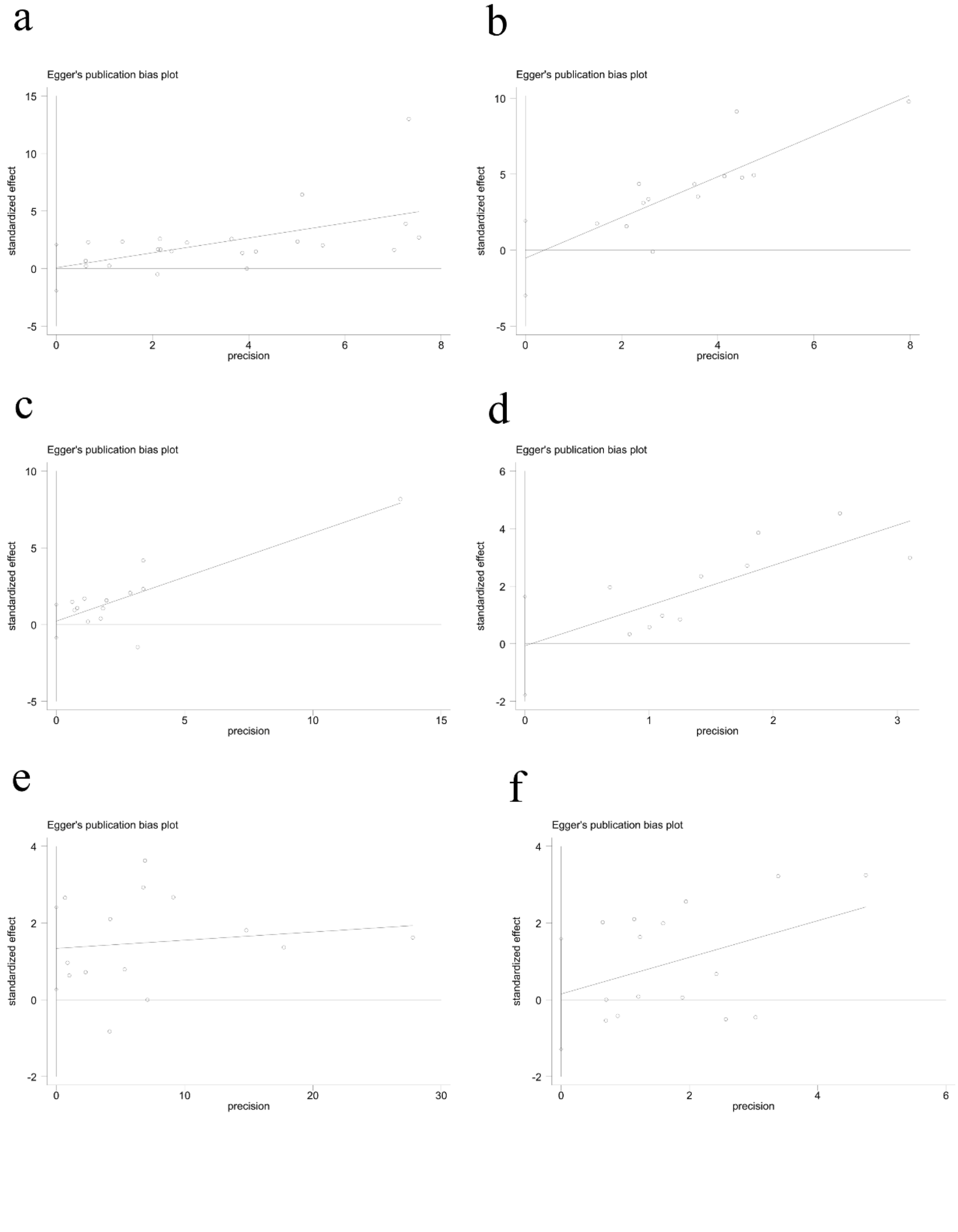

Figure S8. Trim method to evaluate the impact of publication bias on NDI.

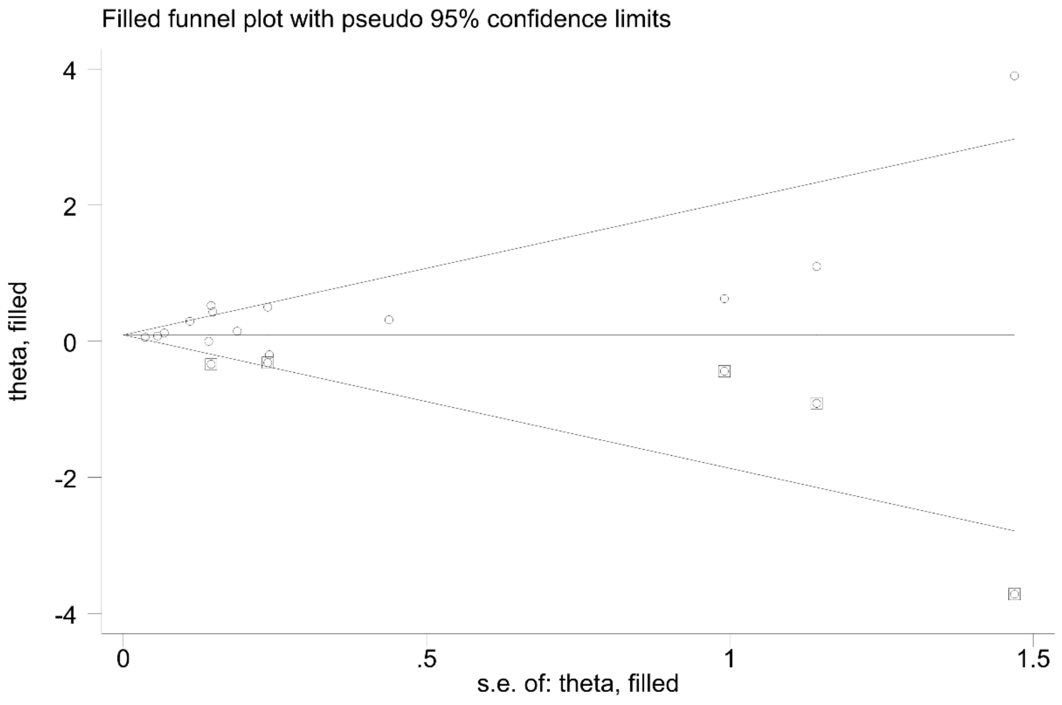

Supplementary Tables

Table S1. PRISMA checklist

Section and Topic	Item #	Checklist item	Location where item is reported
**TITLE**			
Title	1	Identify the report as a systematic review.	Pg. 1
**ABSTRACT**			
Abstract	2	See the PRISMA 2020 for Abstracts checklist.	Pg. 3
**INTRODUCTION**			
Rationale	3	Describe the rationale for the review in the context of existing knowledge.	Pgs. 4 and 6
Objectives	4	Provide an explicit statement of the objective(s) or question(s) the review addresses.	Pg. 6
**METHODS**			
Eligibility criteria	5	Specify the inclusion and exclusion criteria for the review and how studies were grouped for the syntheses.	Pgs. 7-9
Information sources	6	Specify all databases, registers, websites, organisations, reference lists and other sources searched or consulted to identify studies. Specify the date when each source was last searched or consulted.	Pg. 7
Search strategy	7	Present the full search strategies for all databases, registers and websites, including any filters and limits used.	Pg. 7
Selection process	8	Specify the methods used to decide whether a study met the inclusion criteria of the review, including how many reviewers screened each record and each report retrieved, whether they worked independently, and if applicable, details of automation tools used in the process.	Pg. 9
Data collection process	9	Specify the methods used to collect data from reports, including how many reviewers collected data from each report, whether they worked independently, any processes for obtaining or confirming data from study investigators, and if applicable, details of automation tools used in the process.	Pg. 10
Data items	10a	List and define all outcomes for which data were sought. Specify whether all results that were compatible with each outcome domain in each study were sought (e.g. for all measures, time points, analyses), and if not, the methods used to decide which results to collect.	Pgs. 8 and 9
	10b	List and define all other variables for which data were sought (e.g. participant and intervention characteristics, funding sources). Describe any assumptions made about any missing or unclear information.	Pg. 10
Study risk of bias assessment	11	Specify the methods used to assess risk of bias in the included studies, including details of the tool(s) used, how many reviewers assessed each study and whether they worked independently, and if applicable, details of automation tools used in the process.	Pg. 10
Effect measures	12	Specify for each outcome the effect measure(s) (e.g. risk ratio, mean difference) used in the synthesis or presentation of results.	Pg. 10
Synthesis methods	13a	Describe the processes used to decide which studies were eligible for each synthesis (e.g. tabulating the study intervention characteristics and comparing against the planned groups for each synthesis (item #5)).	Pg. 10
	13b	Describe any methods required to prepare the data for presentation or synthesis, such as handling of missing summary statistics, or data	Pg. 10-11

Section and Topic	Item #	Checklist item	Location where item is reported
		conversions.	
	13c	Describe any methods used to tabulate or visually display results of individual studies and syntheses.	Pg. 10
	13d	Describe any methods used to synthesize results and provide a rationale for the choice(s). If meta-analysis was performed, describe the model(s), method(s) to identify the presence and extent of statistical heterogeneity, and software package(s) used.	Pgs. 10-11
	13e	Describe any methods used to explore possible causes of heterogeneity among study results (e.g. subgroup analysis, meta-regression).	/
	13f	Describe any sensitivity analyses conducted to assess robustness of the synthesized results.	Pg. 11
Reporting bias assessment	14	Describe any methods used to assess risk of bias due to missing results in a synthesis (arising from reporting biases).	Pg. 11
Certainty assessment	15	Describe any methods used to assess certainty (or confidence) in the body of evidence for an outcome.	Pg. 10
**RESULTS**			
Study selection	16a	Describe the results of the search and selection process, from the number of records identified in the search to the number of studies included in the review, ideally using a flow diagram.	Pg. 12
	16b	Cite studies that might appear to meet the inclusion criteria, but which were excluded, and explain why they were excluded.	Pg. 12
Study characteristics	17	Cite each included study and present its characteristics.	Pg. 12
Risk of bias in studies	18	Present assessments of risk of bias for each included study.	Pg. 12
Results of individual studies	19	For all outcomes, present, for each study: (a) summary statistics for each group (where appropriate) and (b) an effect estimate and its precision (e.g. confidence/credible interval), ideally using structured tables or plots.	Pgs. 12-16
Results of syntheses	20a	For each synthesis, briefly summarise the characteristics and risk of bias among contributing studies.	Pgs. 12-16
	20b	Present results of all statistical syntheses conducted. If meta-analysis was done, present for each the summary estimate and its precision (e.g. confidence/credible interval) and measures of statistical heterogeneity. If comparing groups, describe the direction of the effect.	Pgs. 12-16
	20c	Present results of all investigations of possible causes of heterogeneity among study results.	/
	20d	Present results of all sensitivity analyses conducted to assess the robustness of the synthesized results.	Pgs. 15-16
Reporting biases	21	Present assessments of risk of bias due to missing results (arising from reporting biases) for each synthesis assessed.	/
Certainty of evidence	22	Present assessments of certainty (or confidence) in the body of evidence for each outcome assessed.	/
**DISCUSSION**			
Discussion	23a	Provide a general interpretation of the results in the context of other evidence.	Pgs. 18-21

Section and Topic	Item #	Checklist item	Location where item is reported
	23b	Discuss any limitations of the evidence included in the review.	Pgs. 20-21
	23c	Discuss any limitations of the review processes used.	Pgs. 22
	23d	Discuss implications of the results for practice, policy, and future research.	Pgs. 18 and 21
**OTHER INFORMATION**			
Registration and protocol	24a	Provide registration information for the review, including register name and registration number, or state that the review was not registered.	Pg. 7
	24b	Indicate where the review protocol can be accessed, or state that a protocol was not prepared.	Pg. 7
	24c	Describe and explain any amendments to information provided at registration or in the protocol.	NA
Support	25	Describe sources of financial or non-financial support for the review, and the role of the funders or sponsors in the review.	Pg. 32
Competing interests	26	Declare any competing interests of review authors.	Pg. 32
Availability of data, code and other materials	27	Report which of the following are publicly available and where they can be found: template data collection forms; data extracted from included studies; data used for all analyses; analytic code; any other materials used in the review.	Pg. 24

Table S2. Baseline characteristics of the 37 studies for meta-analysis

Study	Study type	country	Included infants (centers)	MAX GA	MAX BW (g)	Method of IVH diagnosis	IVH comparison between grade	Severe/Mild outcome	Mild/none outcome	Time to analyze
Treluyer, L.et al.,2023[19]	PC	French	3129(single)	32	…	cranial ultrasound	Severe/Mild; Mild/none	NDI; IQ; CP; epilepsy; visual disability; hearing disability	NDI; IQ; CP; epilepsy; visual disability; hearing disability	at age 5
Reis, Joana Soares.et al.,2023[20]	RC	Portugal	124(single)	32	…	cranial ultrasound	Mild/none	…	CP;auditory deficit;blindness; Gross motor/locomotion; cognition	at 24–36 months of corrected age
Perisset, Alexandra.et al.,2023[21]	RC	Switzerland	509(single)	32	…	cranial ultrasound	Mild/none	NDI; MDI; PDI; CP; visual problems; hearing problems	…	at two years corrected age
Yaghini, O. et al., 2022 [22]	PC	Iran	100(multicenter)	34	VLBW < 1500	brain ultrasonography	Mild/none	…	cognition; communication; receptive language; expressive language; fine motor and gross motor performance	at 8 years
Wang, Y. et al., 2022[8]	PC	China	1079(single)	30	…	cerebral ultrasound	Severe/Mild; Mild/none	CP; MDI < 70; deafness; blindness; neurodevelopmental disability	CP; MDI<70; deafness; blindness; neurodevelopmental disability	at 18–24 months of corrected age
Shah, Vibhuti.et al.,2022[23]	RC	Canada	2327(multicenter)	29	…	cranial ultrasound	Severe/Mild; Mild/none	NDI; CP; congnition/ language/ motor score	NDI; CP; congnition/language/ motor score	at 18 and 24 months of corrected age

Hwang-Bo, Seok.et al., 2022[24]	RC	Korea	191(single)	32	VLBW < 1500	brain sonogram	Severe/Mild	seizure; cognitive score; language score; motor score; socio-emotional score; adaptive behavior score	…	at 18 months of corrected age
Cha, J. H.et al.,2022[25]	RC	Korea	5734(multicenter)	…	VLBW < 1500	cranial ultrasonography,	Mild/none	…	Motor/cognitive/ visual/hearing impairment	aged 12–42 months
Hollebrandse, N. L.et al., 2021[26]	RC	Australia	499(multicenter)	28	…	cranial ultrasonography	Severe/Mild; Mild/none	CP; IQ; executive, academic and motor outcomes	CP; IQ; executive, academic and motor outcomes	at 8 years of age
Bae, Seong Phil.et al.,2021[27]	RC	Korea	240(single)	…	VLBW < 1500	brain USG	Mild/none	…	NDI; CP; cognitive/language/ motor score	at a corrected age of 18–24 months
Shankaran, Seetha.et al.,2020[28]	RC	America	4216(multicenter)	26	…	normal cranial head ultrasounds	Severe/Mild; Mild/none	NDI; CP; cognitive/motor score; hearing/vison impairment	NDI; CP; cognitive/motor score; hearing/vison impairment	18-22 months of corrected age
Scott, T. E.et al., 2020[29]	RC	America	293(single)	32	…	ultrasound	Mild/none	…	cognitive/language/motor scores; CP; seizure; hearing loss	between 24 and 42 months chronologic age
Tu, Yi-Fang.et al., 2019[10]	CC	China	806(multicenter)	32	VLBW < 1500	brain ultrasound	Severe/Mild; Mild/none	epilepsy	epilepsy	5 years of age
Peixoto, Sara.et al., 2018[30]	CC	Portugal	172(single)	34	…	cranial ultrasound	Mild/none	…	CP; NDI; visual impairment; hearing loss	at 24 months of age

Gilard, Vianney.et al., 2018[31]	PC	France	122(single)	< 37	…	cranial ultrasound	Severe/Mild	CP; gross motor function; language development; severe visual impairment; deafness; epilepsy	…	24 months of corrected age
Pfahl, S.et al., 2018[32]	RC	Germany	89(single)	32	…	cranial ultrasound	Severe/Mild; Mild/none	CP; PDI; MDI; NDI; blindness; hearing loss	CP; PDI; MDI; NDI; blindness; hearing loss	at 18–24 months of corrected age
Reubsaet, P.et al., 2017[9]	CC	Netherlands	342(single)	32	…	cranial ultrasound	Mild/none	…	CP; epilepsy; NDI; visual impairment; hearing impairment; cognitive/motor score	at 2 years' corrected age
Wy, P. Ann.et al., 2015[33]	PC	America	985(multicenter）	< 37	2500	cranial ultrasound	Mild/none	…	IQ; cognitive functioning; behavior and academic achievement	at 18 years of age
Radic, Julia A. E.et Al., 2015[34]	PC	Canada	1018(multicenter)	30	…	cranial ultrasound	Severe/Mild; Mild/none	NDI; CP; MDI; blindness; bilateral deafness	NDI; CP; MDI; blindness; bilateral deafness	2 to 3 years of age (corrected age)
Vohr, Betty R.et al., 2014[35]	PC	America	338(multicenter)	< 37	1250	cranial ultrasound	Severe/Mild; Mild/none	CP; IQ; bilateral blind or HL with amplification	CP; IQ; bilateral blind or HL with amplification	at 16 years of age
Bolisetty, Srinivas.et al., 2014[14]	RC	Australia	1472(multicenter)	28	…	cranial ultrasound	Severe/Mild; Mild/none	CP; MDI; NDI; bilateral blindness; hearing loss	CP; MDI; NDI; bilateral blindness; hearing loss	at 2 to 3 years' corrected age

Payne, Allison H.et al., 2013[12]	PC	America	1472(multicenter)	27	…	cranial ultrasound	Severe/Mild; Mild/none	CP;NDI; cognitive/language score; severe visual impairment; deafness	CP;NDI; cognitive/language score; severe visual impairment; deafness	at 18 to 22 months' corrected age
Merhar, S. L.et al., 2012[36]	PC	America	166(multicenter)	…	ELBW<1000	cranial ultrasound	Severe/Mild	PDI; MDI; NDI	…	18–22 months
Klebermass-Schrehof, K. et al.,2012[37]	RC	Germany	471(single)	32	…	cranial ultrasound	Severe/Mild; Mild/none	CP; MDI; NDI; visual impairment; acoustic impairment	CP; MDI; NDI; visual impairment; acoustic impairment	at the age of 5.5 years
Choi, Il Rak.et al., 2012[38]	RC	Korea	49(single)	31	VLBW < 1500	cranial ultrasound	Mild/none	…	MDI; PDI	at a corrected age of 12 months
Broitman, Eduardo.et al.,2007[39]	RC	America	2103(multicenter)	…	ELBW<1000	head ultrasound scanning	Severe/Mild; Mild/none	NDI; MDI; PDI; CP; blindness; deafness	NDI; MDI; PDI; CP; blindness; deafness	at 18 to 22 months corrected age
Patra, K.et al., 2006[40]	PC	America	706(single)	…	ELBW<1000	cranial ultrasound	Mild/none	…	NDI; PDI; MDI; major neurologic abnormality; deafness	at 20 months' corrected age
Ancel, P. Y. et al., 2006[41]	PC	France	1954(multicenter)	32	…	cranial ultrasound	Severe/Mild; Mild/none	CP	CP	at 2 years
Sherlock, R. L.et al., 2005[42]	PC	Australia	298(single)	28	1000	cranial ultrasound	Severe/Mild; Mild/none	CP; IQ; major neurosensory disability	CP; IQ; major neurosensory disability	at 8 years of age
MO'Keefe. et al., 2001[43]	PC	Ireland	68(single)	35	2240	cranial ultrasonography	Severe/Mild	CP; visual acuity <6/60	…	between 12–150 months

Doyle LW,et al., 2000[44]	PC	Australia	424(single)	…	VLBW < 1500	cranial ultrasound	Severe/Mild; Mild/none	CP	CP	at 5 years of age
Bendersky, M.et al., 1995[45]	PC	America	105(single)	35	2000	cranial ultrasound	Severe/Mild; Mild/none	IQ; memory; language; bayley motor score	IQ; memory; language; bayley motor score	at 3 years of age
Landry, S. H.et al., 1993[46]	PC	America	78(single)	34	< 1600	ultrasound or CT scan	Severe/Mild	motor score; IQ	…	at 6, 12,24 and 36 months of age
Vohr, B.et al., 1992[47]	PC	America	112(single)	34	< 1750	cranial ultrasound	Severe/Mild; Mild/none	cognitive Index; perceptual Index; visual-motor development	cognitive Index; perceptual Index; visual-motor development	at 5 years of age
Vohr, B. R.et al., 1989[48]	PC	America	112(single)	34	< 1750	cranial ultrasound	Severe/Mild; Mild/none	PDI; MDI; VER latency; Kohen-Raz subscores; Mullen scores	PDI; MDI; VER latency; Kohen-Raz subscores; Mullen scores	the first 2 years of life
Morales, W. J, 1987[49]	PC	America	303(single)	…	VLBW < 1500	echoencephalogram	Severe/Mild; Mild/none	PDI; MDI	PDI; MDI	at 1 years of age
Ment, L. R.et al.,1985[50]	PC	America	164(single)	…	1250	cranial ultrasound	Severe/Mild; Mild/none	IQ; The Bayley Scales of Infant Development	IQ; The Bayley Scales of Infant Development	at 30 months' corrected age

*PC：prospective cohort study RC：retrospective cohort study CC：case-control study

Table S3. Risk of bias for 34 cohort studies included as per modified Newcastle-Ottawa Scale

Study	Selection				Comparability (2 stars)	Outcome			Total scores
	Representativeness of the exposed cohort	Representativeness of the non-exposed cohort	Ascertainment of exposure	Demonstration that outcome of interest was not present at start of study	Comparability of the exposed cohort and the non-exposed cohort on the basis of the design or analysis	Assessment of outcome	Was follow-up long enough for outcomes to occur	Adequacy of follow-up	
Treluyer, L.et al.,2023[19]	1	1	1	1	2	1	1	0	8
Reis, Joana Soares.et al.,2023[20]	1	1	1	1	2	1	1	1	9
Perisset, Alexandra.et al.,2023[21]	1	1	1	1	1	1	1	1	8
Yaghini, O. et al., 2022[22]	1	1	1	1	2	1	1	1	9
Wang, Y. et al., 2022[8]	1	1	1	1	2	1	1	1	9
Shah, Vibhuti.et al.,2022[23]	1	1	1	1	2	1	1	1	9
Hwang-Bo, Seok.et al., 2022[24]	1	1	1	1	1	1	1	1	8
Cha, J. H.et al.,2022[25]	1	1	1	1	2	1	1	0	8
Hollebrandse, N. L.et al., 2021[26]	1	1	1	1	1	1	1	1	8
Bae, Seong Phil.et al.,2021[27]	1	1	1	1	2	1	1	0	8

Shankaran, Seetha.et al.,2020[28]	1	1	1	1	2	1	1	1	9
Scott, T. E.et al., 2020[29]	1	1	1	1	1	1	1	1	8
Gilard, Vianney.et al., 2018[31]	1	1	1	1	1	1	1	1	8
Pfahl, S.et al., 2018[32]	1	1	1	1	1	1	1	1	8
Wy, P. Ann.et al., 2015[33]	1	1	1	1	2	1	1	1	9
Radic, Julia A. E.et al., 2015[34]	1	1	1	1	2	1	1	1	9
Vohr, Betty R.et al., 2014[35]	1	1	1	1	2	1	1	1	9
Bolisetty, Srinivas.et al., 2014[14]	1	1	1	1	2	1	1	0	8
Payne, Allison H.et al., 2013[12]	1	1	1	1	2	1	1	1	9
Merhar, S. L.et al., 2012[36]	1	1	1	1	2	1	1	1	9
Klebermass-Schrehof, K.et al.,2012[37]	1	1	1	1	2	1	1	1	9
Choi, Il Rak.et al., 2012[38]	1	1	1	1	1	1	1	1	8
Broitman, Eduardo.et al.,2007[39]	1	1	1	1	1	1	1	1	8

Patra, K.et al., 2006[40]	1	1	1	1	2	1	1	1	9
Ancel, P. Y. et al., 2006[41]	1	1	1	1	1	1	1	1	8
Sherlock, R. L.et al., 2005[42]	1	1	1	1	1	1	1	1	8
M O'Keefe.et al., 2001[43]	1	1	1	1	1	1	1	1	8
Doyle LW,et al., 2000[44]	1	1	1	1	1	1	1	1	8
Bendersky, M.et al., 1995[45]	1	1	1	1	2	1	1	1	9
Landry, S. H.et al,1993[46]	1	1	1	1	1	1	1	0	7
Vohr, B.et al,1992[47]	1	1	1	1	1	1	1	0	7
Vohr, B. R.et al,1989[48]	1	1	1	1	1	1	1	1	8
Morales, W. J, 1987[489]	1	1	1	1	1	1	1	0	7
Ment, L. R.et al,1985[50]	1	1	1	1	1	1	1	0	7

Table S4. Risk of bias for 3 case-control studies included as per modified Newcastle-Ottawa Scale

Item		Tu, Yi-Fang.et al., 2019[10]	Peixoto, Sara. et al., 2018 [30]	Reubsaet, P.et al., 2017 [9]
Was the Case Definition and Diagnosis Adequate				
	A. Yes, with independent validation☆	1	1	1
	B. Yes (e.g., from medical records or the doctor's own records)			
	C. No description			
Representativeness of the Cases				
	A. Continuous cases, or the cases are representative cases ☆	1	1	1
	B. Potential for selection biases, or not stated.			
Selection of Controls				
	A. Community controls☆			
	B. Hospital controls	0	0	0
	C. No description			
Definition of Controls				
	A. No history of disease(endpoint)☆	1	1	1
	B. No description of source			
**Comparability（2 points）**				
Comparability of Cases and Controls on the Basis of the Design or Analysis				
	A. Select and analyze controls according to the most important factors☆	1	1	1
	B. Select and analyze controls based on other important factors (such as the second most important factor) ☆	1	0	1
Ascertainment of Exposure				
	A. Reliable records (such as surgical records) ☆	1	1	1
	B. Blind interview (it is unknown who are cases or controls) ☆			
	C. Unblinded interview			
	D. Self-documentation or medical record			

	E. No description			
Same Method of Ascertainment for Cases and Controls				
	A. Yes ☆	1	1	1
	B. No			
No response rate				
	A. The no response rate of the two groups is the same☆	1	1	1
	B. No description			
	C. Response rates vary but reasons are not stated			
Total score		8	7	8
